# Sex-related differences in carpal arch morphology

**DOI:** 10.1371/journal.pone.0217425

**Published:** 2019-05-22

**Authors:** Kishor Lakshminarayanan, Rakshit Shah, Zong-Ming Li

**Affiliations:** 1 Hand Research Laboratory, Department of Biomedical Engineering, Cleveland Clinic, Cleveland, Ohio, United States of America; 2 Department of Chemical and Biomedical Engineering, Cleveland State University, Cleveland, Ohio, United States of America; 3 Department of Orthopaedic Surgery, Cleveland Clinic, Cleveland, Ohio, United States of America; 4 Department of Physical Medicine and Rehabilitation, Cleveland Clinic, Cleveland, Ohio, United States of America; Universidade Federal Fluminense, BRAZIL

## Abstract

The purpose of this study was to investigate the sex-based differences in the carpal arch morphology. Carpal arch morphology was quantified using palmar bowing and area of the arch formed by the transverse carpal ligament. The carpal arch was imaged at the distal and proximal tunnel levels using ultrasonography in 20 healthy young adults (10 women and 10 men). It was found that females had a smaller carpal arch height compared to men at both distal and proximal levels (p<0.05) and smaller carpal arch width only at the proximal level (p<0.05) but not distally. Palmar bowing index, the carpal arch height to width ratio, was significantly smaller in females at the distal level (p<0.05) but not at the proximal level. Carpal arch cross-sectional area normalized to the wrist cross-sectional area was found to be significantly smaller in females at both tunnel levels compared to men (p<0.05). This study demonstrates that females have a smaller carpal arch compared to men with a reduced palmar bowing distally and a smaller arch area at both tunnel levels. The findings help explain the higher incidence of carpal tunnel syndrome in women as a smaller carpal arch makes the median nerve more vulnerable to compression neuropathy.

## Introduction

The carpal tunnel is formed by the transverse carpal ligament (TCL) at its volar boundary and the carpal bones at its medial, lateral, and dorsal boundaries. The tunnel serves as a passageway for the median nerve and digit flexor tendons. The median nerve is situated beneath the TCL and provides motor and sensory function to the hand. The delicate positioning of the median nerve within the tunnel makes it susceptible to compression from area reduction or shape alteration of the TCL-formed carpal arch. Prolonged compression of the median nerve could lead to compression neuropathy known as carpal tunnel syndrome (CTS).

CTS occurrence has a sex propensity with women being 3 times more likely to develop the condition [1]. One possible cause proposed for the higher incidence is that women have a smaller wrist size than men [2]. The smaller wrist size in women also correlates with a relatively smaller carpal tunnel cross-sectional area in women compared to men [3, 4]. However, there were no sex-related differences in the relative carpal tunnel contents area [5, 6]. Specifically, the relative median nerve cross-sectional area was not significantly different between the sexes [6]. The reduced available space for the tunnel contents in women could increase the likelihood of the median nerve getting compressed against the TCL. With median nerve being in close proximity to the TCL, investigating the sex-related differences in TCL-formed carpal arch morphology could provide insight into the higher incidence of CTS in women.

In addition to sex-related differences in carpal tunnel area, women have less compliant carpal tunnels compared to men [7]. Moreover, women have been shown to have a less elastic TCL than men [8] which may contribute to the reduced carpal tunnel compliance in women. Based on these evidence, it is possible that women have a predisposition for a reduced palmar bowing of the TCL compared to men. Palmar bowing of the TCL has been shown to increase to accommodate for enlargement of carpal tunnel contents [9], especially in CTS cases [10–12]. A less elastic TCL might not be able to palmarly bow adequately to accommodate for the reduction in available space for contents or elevated pressure within the tunnel. Consequently, a reduced palmar bowing in tandem with a smaller TCL-formed carpal arch in women could make them more susceptible to median nerve compression by the TCL. However, to date, little is known about the sex-related differences in the carpal arch.

The purpose of this study was to determine whether the TCL-formed carpal arch is different between healthy women and men at the distal and proximal carpal tunnel. The carpal arch was quantified by palmar bowing and area of the carpal arch. It was hypothesized that women would have smaller palmar bowing and arch area compared to men, especially in the distal tunnel.

## Methods

### Subjects

Twenty healthy young adults (10 women and 10 men) were recruited for the study. The age, weight, height, and BMI of the females were 28.4±3.9 years, 56.3±9.0 kg, 154.8±16.7 cm, and 21.7±2.0 kg/m^2^ respectively. The age, weight, height, and BMI of the males were 26.1±2.4 years, 66.8±5.6 kg, 175.4±6.4 cm, 22.2±2.0 kg/m^2^, respectively. No subjects had any history of upper limb injury or musculoskeletal or neurologic disorders. Wrist width and depth were also collected for calculation of wrist cross-sectional area. All subjects gave informed written consent, and the study received ethical approval from the Cleveland Clinic’s Institutional Review Board.

### Collection of ultrasound videos of the carpal tunnel

**Fig 1:**
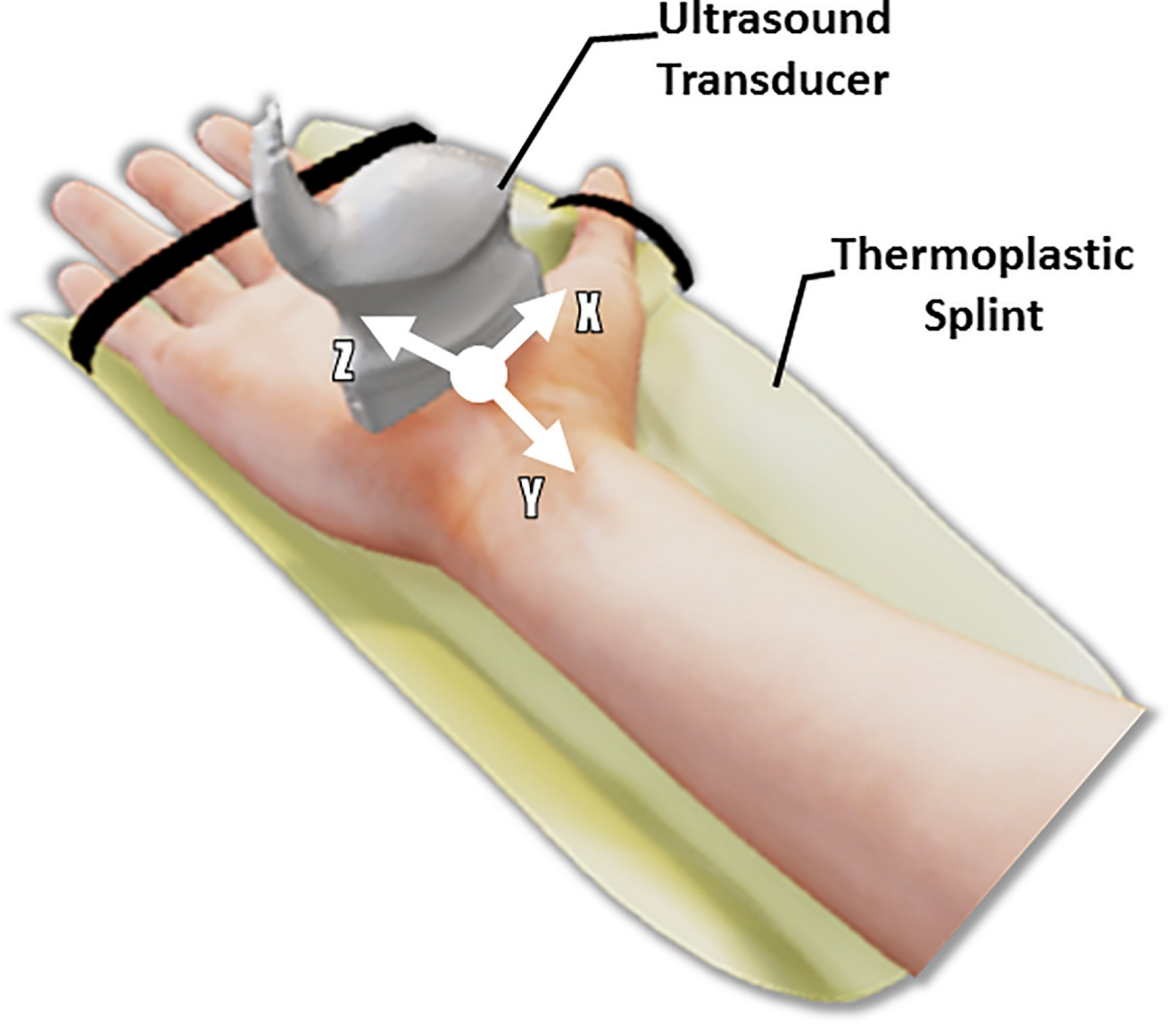
Experimental setup for ultrasound imaging.

Subjects were asked to sit next to a testing table and place their right hand on the testing table with the shoulder abducted 30° and the elbow flexed 90°. The hand and wrist were then stabilized by a thermoplastic splint in a supine and anatomically neutral position (Fig 1). Velcro® straps were used to secure the forearm, stabilize the four fingers in extension, and position the thumb in a naturally abducted position.

An ultrasound system with a 18L6 HD linear array probe (Acuson S2000, Siemens Medical Solutions USA, Mountain View, CA, USA) was utilized for data collection. Ultrasound video was collected at the distal or proximal tunnel region by randomly and continuously translating and rotating the ultrasound probe along and around the X (lateral) and Z (elevation) axes (Fig 1). Each ultrasound scanning video lasted 60 seconds at a rate of 30 Hz, resulting in 1800 ultrasound frames. The vast number of frames ensured that the targeted distal or proximal cross sections of the carpal tunnel were embedded in the video. Three trials of ultrasonographic video collection were conducted at each tunnel level for each subject.

### Processing of ultrasound videos

Each ultrasound video was processed by a custom LabVIEW program to output the best frame containing the target cross-sectional images at the distal and proximal carpal tunnel. Each frame of the ultrasound video was compared against a predetermined reference frame for the distal or proximal tunnel by the custom pattern recognition algorithm to determine the single frame in the video that best matched with the reference frame. The algorithm performed image feature matching with selected regions of interest (ROIs) of containing specific anatomical landmarks. For the distal tunnel reference frame, ROIs were established around anatomical features for the hamate, trapezium, and most ulnar aspect of the thenar muscle attachment to the TCL. Each ROI served as a template for pattern tracking of each frame in the video. The three-match scores by the three ROIs for each frame was summed, and the video frame with the maximal summed score was output as the identified image for the cross-section of the distal tunnel. The identified image was exported by the program as a JPEG file. Among the three identified images for the three videos, the investigators selected a prime image that has the most distinctly visible TCL boundaries for further analyses. The prime image for the proximal carpal tunnel was obtained in a similar process except that two ROIs were established around the locations where the TCL attached to the pisiform and scaphoid.

### Outcome measures

**Fig 2:**
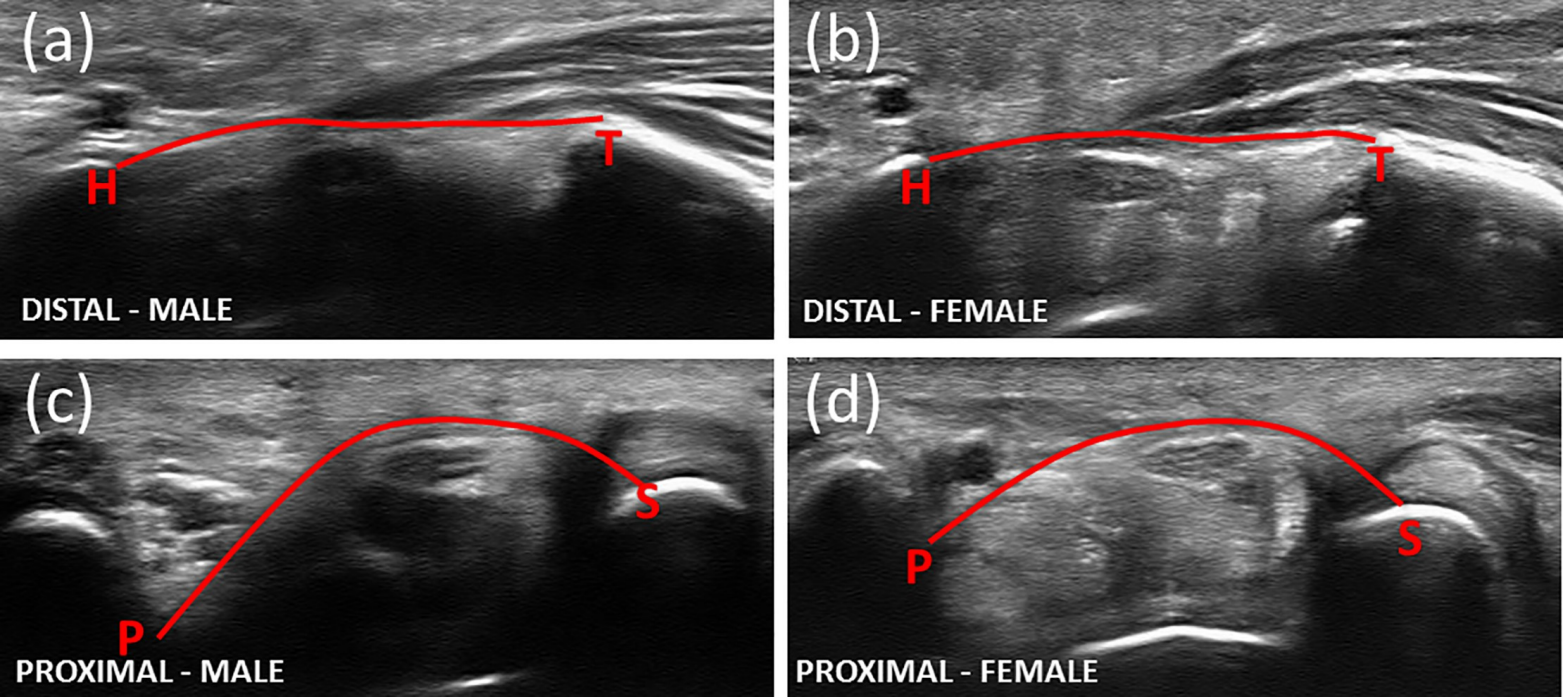
Ultrasound images at each tunnel level (distal and proximal) for a representative male and female subject. The images reflect the selection made in the custom LabVIEW code for the traced TCL (solid line) and the landmark of interest, specifically the hook of hamate (H), ridge of the trapezium (T), pisiform (P), and scaphoid (S).

For each distal and proximal tunnel prime image, the volar boundary of the TCL was manually traced along with the osseous attachment points (ridge of the trapezium and hook of the hamate at distal tunnel and pisiform and scaphoid at proximal tunnel) using another custom LabVIEW program (Fig 2). The tracing was facilitated by magnifying the image when needed. The traced TCL boundary and osseous insertion points were exported as calibrated image coordinates in mm for calculation of carpal arch width, height, and area. Arch width at distal or proximal tunnel was defined as the distance between the respective osseous attachment points of the TCL, and arch height was obtained as the maximal perpendicular distance of the TCL boundary points to the line along the arch width. The arch area was calculated using the integral of the TCL boundary over the arch width. Furthermore, the arch height was normalized with respect to the arch width as a palmar bowing index (PBI), and the arch area was normalized with respect to the wrist cross-sectional area which was approximated as an ellipse with major and minor axes represented by wrist width and thickness. The TCL tracing on the same image were performed three times and associated outcome data were averaged for statistical analyses.

### Statistical analysis

Student’s t-tests were used to compare sex differences in age, body weight, body height, BMI and wrist size. Two-way mixed ANOVAs were used to determine the effect of sex (female and male) and location (distal and proximal) on the dependent variables of the arch height, width, PBI, arch area, and normalized arch area. Post-hoc Bonferroni t-tests were used for all pairwise comparisons. Statistical analyses were performed using SigmaStat 4.0 (Systat Software Inc, San Jose, CA, USA). An α level of 0.05 was considered for statistical significance.

## Results

There were no significant sex differences in age (p = 0.12) and BMI (p = 0.61), but the males had greater body weight (p<0.01), body height (p<0.01), wrist width (p<0.01), and wrist thickness (p<0.05) than females. Sample ultrasound images at the distal and proximal tunnels are shown in Fig 2. In general, females had a smaller carpal arch than men, specifically a reduced palmar bowing and smaller arch area.

**Fig 3:**
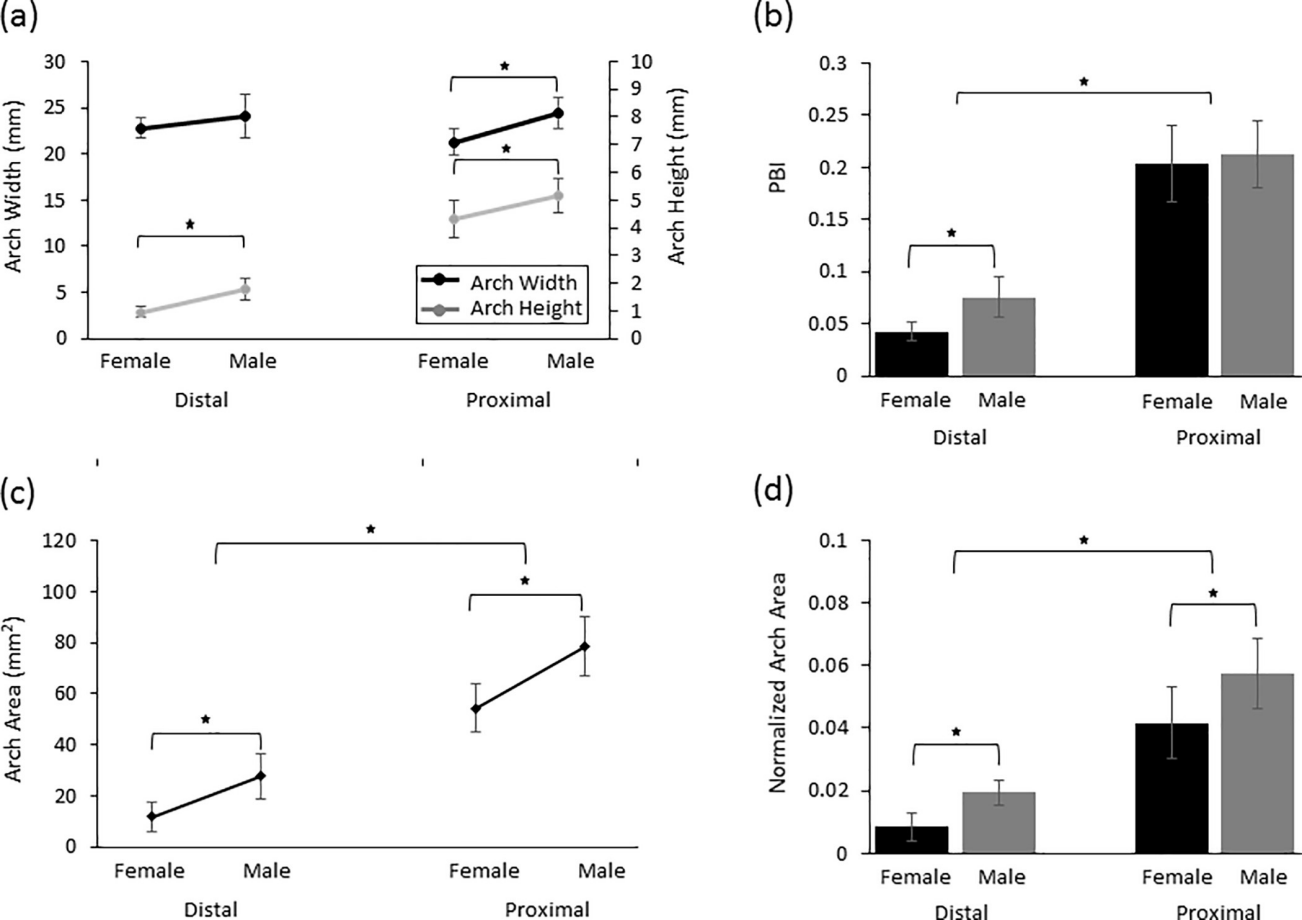
The (a) arch width and height, (b) palmar bowing index, (c), arch area, and (d) normalized arch area at the two tunnel levels (distal and proximal) for the two sexes (female and male). * p<0.05.

Arch height was significantly affected by the factors of sex (p<0.001) and location (p<0.001, Fig 3A), and their interaction was not significant (p = 0.918). Females had significantly smaller arch height compared to men at both locations (p<0.05). At the distal tunnel, the arch height (1.0±0.2 mm) for females was significantly smaller than (i.e. 55.1% of) the arch height (1.8±0.4 mm) for males (p<0.05). At the proximal tunnel, females had an arch height of 4.3±0.7 mm, which was significantly smaller than (i.e. 83.5% of) the arch height of 5.1±0.6 mm for males (p<0.05). The arch heights at the distal tunnel were significantly smaller than that at the proximal tunnel for females (p<0.05) and males (p<0.05), with the distal arch heights as 22.8±4.8% and 35.0±8.9% of the proximal arch heights for females and males, respectively.

Arch width was significantly affected by the factor of sex (p<0.05) but not location (p = 0.172; Fig 3A). The interaction between sex and location also significantly affected the arch width (p<0.05). The arch width at the distal level (22.8±1.1 mm) for females was not significantly different from the arch width (24.1±2.3 mm, p = 0.09) for males. At the proximal level, females had significantly smaller arch width compared to (i.e. 87.0% of) men (21.2±1.4 mm vs. 24.4±1.6 mm, p<0.05). The arch widths at the distal tunnel were significantly larger than (i.e. 107.3±3.9% of) that at the proximal tunnel for females (p<0.05). In males, arch widths at the distal tunnel were not significantly different from that at the proximal tunnels (p = 0.58).

PBI as the arch height normalized with respect to the arch width was found to be significantly affected by the factors of sex (p<0.05) and location (p<0.001), and their interaction was not significant (p = 0.154) (Fig 3B). PBI was significantly smaller in females compared to males at the distal tunnel (p<0.05) but not the proximal tunnel (p = 0.465). At the distal tunnel, females had a significantly smaller PBI of 0.04±0.01 than (i.e. 56.0% of) the PBI for men which was 0.08±0.02 (p<0.05). At the proximal tunnel, the PBI was not significantly different between the sexes (0.20±0.04 for females and 0.21±0.03 for males, p = 0.465). The distal tunnel PBI was significantly smaller than the proximal tunnel for both the sexes, with females and males having a distal PBI as 21.3±4.6% (p<0.05) and 36.3±11.6% (p<0.05) of the proximal PBI, respectively.

Arch area and normalized arch area were both significantly affected by the factors of sex (p<0.001) and location (p<0.001) (Fig 3C and 3D). Sex and location interaction did not significantly affect either the arch area (p = 0.363) or the normalized arch area (p = 0.052). At the distal tunnel, the arch area for females (11.6±5.7 mm^2^) was significantly smaller than (i.e. 42.1% of) the arch area (27.6±8.9 mm^2^) for males (p<0.05). The proximal tunnel arch area for females (54.3±9.6 mm^2^) was significantly smaller than (i.e. 69.2% of) the arch area (78.5±11.5 mm^2^) for males (p<0.05). The arch areas at the distal tunnel were 20.4±11.3% (p<0.05) and 35.2±10.5% (p<0.05) of the areas at the proximal tunnel for females and males, respectively.

Females and males had normalized arch areas of 0.008±0.004 and 0.019±0.004, respectively, at the distal tunnel (Fig 3D). Females had a significantly smaller normalized arch area than (i.e. 41.2% of) males (p<0.05) at the distal level. At the proximal tunnel, the normalized arch area for females (0.04±0.01) were significantly smaller than (i.e 71.9% of) the normalized arch area (0.06±0.01) for males (p<0.05). The distal tunnel normalized arch area was significantly smaller compared to the proximal tunnel for both females and males (p < 0.05).

## Discussion

In this study, we used an imaging processing algorithm to automatically identify the targeted cross sections of the distal and proximal carpal arch. The algorithm eliminated operator dependency to find the arch cross sections that contained the anatomical configuration for manual tracing of the TCL volar boundary. The imaging protocol and automated algorithm demonstrate using ultrasonography as a low-cost alternative to high-resolution MRI for the examination of the TCL-formed carpal arch. The ultrasonographic method can be applied to clinical studies to understand morphological changes of carpal arch in pathological condition (e.g. CTS).

The evaluation of the TCL-formed carpal arch and their differences between the sexes at the distal and proximal tunnels has revealed several findings. The main finding is that females had a smaller palmar bowing than men. The palmar bowing (arch width-to-height ratio) at the distal tunnel in females was approximately half of that in males, although sex-related difference for palmar bowing was less pronounced at the proximal tunnel. Our results are consistent with the findings of Monagle et al. [6] who reported a reduced palmar bowing in females compared to men at the distal tunnel level, but not at the proximal tunnel. The reduced palmar bowing in females could be associated with the less compliant carpal tunnels in women compared to men [7]. The TCL being less elastic [8] but of similar thickness in women compared to men [13] may explain the reduced tunnel compliance and a reduced palmar bowing in women than men. Additionally, the more pronounced reduction in palmar bowing at the distal tunnel compared to the proximal may be attributed by the narrower distal tunnel [14, 15], TCL being thicker distally [13, 14, 16] and the lower carpal arch compliance at the distal tunnel [17].

Our study also showed that females had a smaller carpal arch cross-sectional area compared to men for either distal or proximal tunnel. This sex difference was still valid with the normalization of carpal arch area with respect to wrist size to control the potential confounding factor that females tend to have a smaller wrist. Previous studies have shown that women have smaller carpal tunnel cross-sectional area compared to men at both distal and proximal ends of the tunnel [3, 4]. Our study extended the previous findings of smaller carpal tunnel area in women compared to men as reported to the sex-related differences in carpal arch. The smaller carpal arch area at both distal and proximal tunnels combined with a reduction in palmar bowing in females compared to males show that women have a small and disproportionate TCL-formed arch. The small and disproportionate carpal arch observed in our study further our insights into CTS propensity in females. Our findings show that not just size difference but also structural peculiarities inherent to the female anatomy may make women more susceptible to CTS than men.

The small and disproportionate carpal arch morphology in females compared to males could possibly be caused by females having a small [2] yet disproportionate wrist (wrist depth-to-width ratios) [18] compared to men [19]. Disproportionately smaller wrist is associated with increased median nerve latency in CTS patients [20]. Previous studies have shown that although women had smaller carpal tunnel cross-sectional areas than men at both distal and proximal tunnels, the median nerve cross-sectional area at the distal tunnel was not different between the sexes [4, 6]. With smaller carpal tunnel size, the TCL would ideally bow more palmarly to accommodate the carpal tunnel contents. In contrast, our results showed a pronounced reduction in palmar bowing of the TCL in females at the distal level, which reduces the available space for the carpal tunnel contents including the median nerve. With median nerve being proximal to the TCL and having no sex-related difference in nerve area at the distal tunnel, the reduced arch area and palmar bowing in females could lead to the median nerve getting compressed against the TCL, leading to nerve entrapment. In conclusion, females having reduced palmar bowing and smaller arch area than men, especially at the distal narrower end of the carpal tunnel might play a role in the higher incidence of CTS in women.

S1

## Supporting information

S1 File Subject-specific information and carpal arch morphology for females (Table A) and males (Table B) that underlie the main findings in this study. (XLSX)
